# From Tradition to Future in Veterinary Public Health Education: Students’ Attitudes, Anxiety and Experiences With AI‐Supported Learning

**DOI:** 10.1002/vms3.71056

**Published:** 2026-06-23

**Authors:** Selçuk Alan, Gönül Damla Büyük, Seda Çavuş Alan

**Affiliations:** ^1^ Department of Food Hygiene and Production, Faculty of Veterinary Medicine Kafkas University Kars Türkiye; ^2^ Department of History of Veterinary Medicine and Deontology, Faculty of Veterinary Medicine Kafkas University Kars Türkiye

**Keywords:** anxiety, artificial intelligence, ChatGPT, perceptions, veterinary public health

## Abstract

**Background:**

Artificial intelligence (AI) has the potential to reshape learning processes, especially through tools such as ChatGPT.

**Objectives:**

This study aims to evaluate veterinary students’ attitudes towards AI‐supported veterinary public health education applications, their anxiety levels and their experiences regarding the education process.

**Methods:**

The study adopted a mixed method design in which quantitative and qualitative methods were used together. The study was conducted with final‐year veterinary students (*n* = 60) enrolled in the Veterinary Public Health course. This study consisted of four phases: (1) the Artificial Intelligence Anxiety Scale (AIAS) was administered as a pre‐test, (2) traditional face‐to‐face training was given, (3) the same topic was repeated with a ChatGPT‐based AI‐supported exercise and (4) after the trainings, the AIAS was administered again and post‐test was conducted and a questionnaire consisting of closed and open‐ended questions was administered to the students. Factor analysis revealed that the scale had high internal consistency (Cronbach's *α* > 0.90).

**Results:**

Analysis of the scale scores revealed that veterinary students exhibited moderate anxiety towards AI and that AI‐focused education did not significantly alter this anxiety in the short term. In the content analysis of qualitative data, students stated that they benefited from the aspects of AI such as fast access to information, practicality and time‐saving; the same time, they expressed concerns about ethical concerns, information reliability and professional role change.

**Conclusion:**

Overall, the findings indicate a duality in students’ potential attitudes towards AI. These findings indicate the applicability of AI‐based educational practices in the context of veterinary public health education and point to the need for multidimensional evaluation of student attitudes.

## Graphical abstract

Veterinary students were found to have moderate anxiety towards artificial intelligence (AI) and ChatGPT‐supported education did not change this anxiety level in the short term. While students reported benefiting from AI's advantages such as fast access to information, practicality and time‐saving, they simultaneously expressed concerns about ethical issues, information reliability and professional role changes. The findings revealed that students exhibited dual attitudes towards AI and that AI‐based applications in veterinary public health education require multidimensional evaluation.

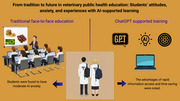

## 1 Introduction

Artificial intelligence (AI) has been increasingly integrated into the educational landscape. In medical education, AI is utilized in virtual simulations for clinical decision‐making, radiology training for image interpretation and adaptive systems that tailor content to individual needs (Sriram et al. 2025). Similarly, dental education has leveraged AI‐based systems for radiograph interpretation, orthodontic treatment planning and simulated patient interactions (Yüzbaşıoğlu 2021). Furthermore, nursing education has incorporated AI‐supported virtual patients to provide opportunities for safe practice and real‐time feedback (Bahari et al. 2025). These applications demonstrate that AI can play a supportive role in health education by enhancing student engagement, improving diagnostic reasoning and complementing traditional teaching approaches (Busch et al. 2024).

AI technologies have permeated various healthcare fields, including veterinary medicine, where they are increasingly integrated into professional practices. However, this rapid progress has been met with both excitement and anxiety (Yüzbaşıoğlu 2021; Worthing et al. 2024). Attitudes about how AI will affect daily human life vary widely between optimistic and pessimistic approaches. Pessimistic approaches to AI include concerns that AI will replace humans in many sectors, while optimistic views point out that people with AI support will benefit more from future technological developments (Morrow et al. 2023). Studies conducted especially with students studying in the field of healthcare show that the lack of knowledge about AI in students leads to concerns that professional roles will narrow and the future position of their professions will become uncertain. It is stated that this situation may cause students to experience decision‐making difficulties in the career planning process (Teng et al. 2022). Despite all these concerns, the application areas of AI technologies in healthcare continue to expand rapidly. From clinical processes, such as the interpretation of radiographic images and contribution to diagnostic processes, to scientific article writing and language translation, the use of AI in many academic applications is increasing (Worthing et al. 2024; Appleby and Basran 2022). The widespread use of AI in veterinary medicine practice has made it imperative for veterinary medicine education to keep pace with this transformation and to provide veterinary medicine students with AI literacy. Consequently, it is imperative for veterinary education to provide students with AI literacy to prepare them for future professional environments (Busch et al. 2024; Jiang et al. 2017).

The veterinary profession extends beyond animal health, playing a vital role in protecting public health. In this context, the Veterinary Public Health (VPH) curriculum covers zoonotic disease control, food safety and environmental health. Furthermore, understanding the relationship between human and animal health—including the critical role of animal models in translational medical research—is essential for assimilating the ‘One Health’ concept (Choudhary 2025).

In addition to traditional theoretical knowledge expression, current technologies such as AI can also be utilized in the effective conduct of VPH education. As a natural language processing tool, ChatGPT facilitates access to information by providing real‐time feedback (Allam et al. 2023). From this perspective, ChatGPT can be a potential auxiliary tool in veterinary medicine education by functioning as a guide in student‐oriented learning processes as it facilitates access to information. In the literature review, although the number of studies using ChatGPT or similar AI tools in veterinary medicine curriculum is limited and predominantly focused on foundational disciplines such as anatomy through virtual models, there is no study that uses AI technology in VPH course (Choudhary et al. 2023; Choudhary et al. 2025; Choudhary and Sarkar 2025; Kuzminsky et al. 2023).

The AI tool used in this study was ChatGPT, a large language model developed by OpenAI. Although not specifically designed for educational purposes, ChatGPT has been increasingly used in academic contexts as a supportive tool for information retrieval, interactive learning and critical discussion. In this study, ChatGPT was integrated into the VPH course to explore its potential contribution to students’ learning experiences.

Although AI applications are increasingly integrated into medical and health education, their use in veterinary education remains limited. Specifically, there is a lack of empirical data regarding how veterinary students perceive AI‐supported learning tools and the subsequent impact on their attitudes and anxiety levels. Therefore, the aim of this study was to evaluate veterinary students’ attitudes, perceptions and anxiety levels regarding the use of ChatGPT‐supported education in the context of VPH. By comparing a traditional teaching method with a ChatGPT‐based exercise, this study sought to explore students’ experiences, perceived advantages and concerns and the potential impact of AI on their learning process. Based on the existing literature, we hypothesized that the integration of ChatGPT‐supported exercises into the VPH course would positively influence students’ attitudes towards AI and reduce their anxiety levels. Furthermore, we expected that students would perceive both advantages (fast access to information, practicality and time‐saving) and concerns (ethical issues, reliability and professional role change) regarding the use of AI in education.

## 2 Materials and Methods

Informed consent was obtained from all participants prior to the study. To ensure confidentiality, the responses were kept anonymous. Each participant was assigned a random number, and the analyses were carried out based on these codes. The researchers did not have access to information linking individual responses to specific students.

### 2.1 Study Design

**FIGURE 1:**
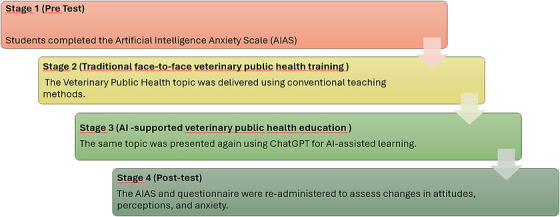
Study design and workflow of the research process.

Veterinary education in (anonymized country) lasts 5 years. The participants in this study were senior (5th‐year) students enrolled in the VPH course, which is included in the curriculum during the final year of education. This study, conducted with veterinary students, consisted of four main stages and is shown in Figure 1. The topic of ‘Pathogenic Microorganisms that can be found in Ready‐to‐Eat Foods’ was taught to the students first using a traditional method and then with a ChatGPT‐supported method. Following the teaching sessions, students’ opinions on the topic were collected through both closed‐ and open‐ended questions. In addition, changes in students’ anxiety levels towards AI were examined.

#### 2.1.1 Stage 1: Pre‐Test

At the beginning of the study, students were administered the Artificial Intelligence Anxiety Scale (AIAS) (Wang and Wang 2019) to measure their initial anxiety levels towards AI. The results were recorded as AIAS‐First. A pilot study was conducted with five students to evaluate the applicability of the scale.

#### 2.1.2 Stage 2: Traditional Face‐to‐Face VPH Training

The students were given a traditional VPH lecture of about 20 min by a researcher with a PhD in Food Hygiene and Technology, which is a discipline of veterinary medicine focusing on food safety and public health. The following topics were covered in the lecture:

- Pathogenic microorganisms that may be present in ready‐to‐eat foods, according to European Union and local regulatory frameworks.
- Pathogens that may be encountered especially in products such as hamburgers, meatballs and doner (which students also consume frequently).
- Morphological characteristics of *Listeria monocytogenes*, *Salmonella* spp. and their importance for public health.
- Hygiene measures to be taken at home to protect against these pathogens.

#### 2.1.3 Stage 3: AI‐Supported VPH Education

After the traditional training, the same topic was presented to the students again, this time using ChatGPT. The programme (GPT‐4, OpenAI) was used in its publicly available form and was not specifically trained or fine‐tuned for this study. Before the study began, the researchers tested the programme with sample prompts to ensure that it could generate coherent and relevant responses on VPH topics. During the session, ChatGPT was instructed to provide responses tailored for students, and only questions related to VPH were posed. The interaction was carried out live in the classroom by projecting ChatGPT onto a screen, where students directly observed the questions and answers in real time. The researcher did not provide any interpretation or commentary but only typed the questions into ChatGPT. The questions posed to ChatGPT are as follows:

*Question 1. What are the pathogenic microorganisms that can be found in ready‐to‐eat foods, according to the European Union and local regulatory frameworks?*
*Question 2. What are the pathogenic microorganisms that can be found in ready‐to‐eat foods such as hamburgers, meatballs and doner kebabs?*
*Question 3. What are the morphological characteristics of these pathogens, especially L. monocytogenes and Salmonella spp. and why are they risky for public health?*
*Question 4. What precautions should a person who prepares and cooks ready‐to‐eat foods in the kitchen take to protect against L. monocytogenes and Salmonella spp.?*

#### 2.1.4 Stage 4: Post‐Test

After the completion of the trainings, the questionnaire form including the Introductory Information Form and AIAS was administered to the students again and this application was evaluated as a post‐test. AIAS results were recorded as AIAS‐Last. Thus, it was analysed whether there was any change in students’ anxiety levels towards AI compared to the pre‐training period.

### 2.2 Data Collection Methods

***AI anxiety scale (AIAS)***: It is a 16‐item scale developed by Wang and Wang (2019) and adapted into the local language by Akkaya et al. (2021). The items are scored on a 5‐point Likert‐type rating scale (from 1 = strongly disagree to 5 = strongly agree). Scores that can be obtained from the scale vary between 16 and 80. Higher scale scores indicate greater levels of AI‐related anxiety (Wang and Wang 2019). The internal consistency of the localized version of the scale is 0.937 and explains 76.88% of the total variance.

***Introductory information form***: Three closed‐ended questions (What is your gender? Have you used AI before? Do you think that AI course should be added to your education curriculum?), five Likert‐type statements (‘The use of AI in veterinary medicine will increasingly cause legal and ethical conflicts’, ‘I think AI will reduce the need for veterinarians’, ‘I think AI can be used as an educational tool in veterinary public health education’, ‘I plan my career according to developments in AI’, and ‘I can apply AI programmes about foodborne diseases’) and an open‐ended question (After using AI as part of a Veterinary Public Health course, how would you evaluate AI? [a] What are the benefits? [b] What are the risks? [c] Which metaphor can you compare this situation to?).

### 2.3 Participants and Data Collection

The study population consisted of final‐year veterinary students (*n* = 90) enrolled in the course ‘VPH’ at (anonymized university) Faculty of Veterinary Medicine (Student Information System, 2024). The study included 60 students who voluntarily agreed to participate. At the beginning of the questionnaire form, there was a section informing the participants about the purpose of the study. It was clearly stated that no personal information would be requested from the participants, that the study would be conducted entirely on a voluntary basis, and that participants could leave the study without any justification if they wished. It was also committed that the data obtained would only be used for scientific purposes. The data collection process was conducted face‐to‐face on 17 March 2025, outside of the students’ class hours.

In the study, mixed method was used; both qualitative and quantitative data were collected. While the quantitative method enables the phenomena and events to be expressed, measured and generalized numerically, the qualitative method helps to understand and reveal the subject in detail, as it allows the participants to explore the ways of understanding and interpreting these phenomena and events (Plano Clark 2016). In the study, questions 1 to 8 were closed‐ended, while question 9 was an open‐ended question consisting of three sub‐headings.

### 2.4 Data Analysis

Exploratory factor analysis (EFA) was performed using the JAMOVI 2.2.5 statistical package programme to evaluate the structure of the measurement tool used in the study and its suitability for the sample. The suitability of the data for factor analysis was assessed using Bartlett's test of sphericity and the Kaiser–Meyer–Olkin (KMO) measure. A Bartlett's test of sphericity with *p* < 0.001 and a KMO value ≥ 0.50 indicate that the data are suitable for EFA. In EFA, varimax rotation was used to reveal the factors. Only items with factor loadings of 0.40 and above were included in the study. In determining the number of factors, the scree plot was examined and factors with eigenvalues greater than 1 were retained. Internal consistency was measured by Cronbach's *α*. Demographic data were analysed using SPSS 22 software. The results were evaluated at 95% confidence interval (*p* < 0.05 level). Percentage and frequency analyses were used for demographic data. The distribution of the data was evaluated by Kolmogorov–Smirnov, mean, standard deviation, skewness, kurtosis values and histogram, boxplot and Q–Q plots and it was seen that the data were normally distributed. Independent *T*‐test and ANOVA test were used in the scale and independent variable questions. Paired sample *T*‐test was used to compare the pre‐test and post‐test scores of the AIAS scale in terms of significance.

‘Content analysis’ method was used to analyse the qualitative questions in the questionnaire form (Baş and Akturan 2008). Codes and themes were created in order to reveal patterns from the views of the participants and the data were evaluated accordingly. During this process, it was taken into consideration that the data were both independent expressions in themselves and constituted parts of a holistic structure. In addition, the ‘phenomenological design’ was used to investigate the individual universe and to understand how the participants perceived the subject of AI, the meaning they attributed to it and what they thought about its integration into VPH education. Participants were asked *‘After using AI as part of a VPH course, how would you evaluate AI? What are the benefits? What are the risks? Which metaphor can you liken this situation to?’* and the answers were evaluated by content analysis method.

## 3 Results

### 3.1 Quantitative Findings (AIAS Scale and Statistical Analyses)

Initially 61 people participated in the study. However, in the middle of the study period, one student left the study due to health reasons, stating that he/she did not want to continue the study. Therefore, the analyses were conducted on a total of 60 participants. A total of students completed both the pre‐test and post‐test phases of the study. Table 1 presents the mean score of the AIAS‐First, administered prior to the AI‐supported training, was 43.73 (Cronbach's *α* = 0.919), while the mean score after the training (AIAS‐Last) was 42.26 (Cronbach's *α* = 0.945). When AIAS‐First and AIAS‐Last scores were compared, the difference between them was not statistically significant (*p* = 0.517).

**TABLE 1:** Cronbach's *α* values and mean total scores of the Artificial Intelligence Anxiety Scale (AIAS), administered before (AIAS‐First) and after (AIAS‐Last) the AI‐supported training. Higher scores indicate greater anxiety.

Variables	Cronbach's *α*	Score	Paired sample *T*‐test
AI Anxiety Scale (AIAS‐First)	0.919	43.733	*p* = 0.517
AI Anxiety Scale (AIAS‐Last)	0.945	42.266	

**FIGURE 2:**
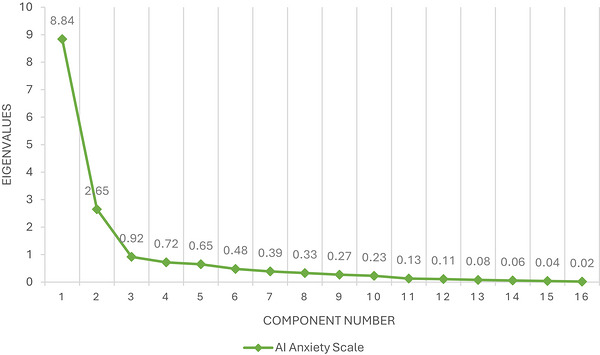
Scree plot chart of exploratory factor analysis (EFA) results for the Artificial Intelligence Anxiety Scale (AIAS‐Last).

An EFA was performed on the second AIAS (AIAS‐Last) data administered at the end of the trainings to determine the factor structure of the scale in the sample of veterinary students. Before the analysis, KMO and Bartlett's test of sphericity were performed to reveal the suitability of the data for factor analysis. Figure 2 presents the scree plot of eigenvalues obtained from this analysis. As a result, KMO value was calculated as 0.851 and Bartlett's test of sphericity value as *p* < 0.001. Then, EFA was performed with varimax rotation method. As a result of the analysis, scree plot chart and eigenvalues were analysed. For AIAS‐Last, two factors with eigenvalues above 1 explained 71.9% of the variance.

Table 2 summarizes the demographic characteristics and prior AI experiences of the students. Demographic analysis showed that 68.3% of participants were male and 31.7% were female. A majority of students reported prior AI experience, with only 3.3% having never utilized AI tools. Notably, 72.9% of participants advocated for the integration of AI into the veterinary curriculum.

**TABLE 2:** Demographic characteristics and prior experiences with artificial intelligence of participating veterinary students (*n* = 60).

	Number	Percentage (%)
Gender		
Female	19	31.7
Male	41	68.3
Have you used artificial intelligence before?		
Never used	2	3.3
2–3 times	13	21.7
I use it occasionally	35	58.3
I use it all the time	10	16.7
Do you think an AI course should be added to your education curriculum?		
Yes	43	72.9
No	16	27.1

When Likert‐type questions were analysed, Table 3 presents the mean scores (±SD) of students’ responses to different statements on AI in VPH education. The statement with the highest agreement was ‘I think AI will reduce the need for veterinarians’ (3.70 ± 1.10), followed by ‘I plan my career according to developments in AI’ (3.60 ± 0.94). Conversely, the lowest scores were observed for ‘I can apply AI programmes about foodborne diseases’ (2.25 ± 0.81) and ‘I think AI can be used as an educational tool in VPH education’ (2.38 ± 1.05).

**TABLE 3:** Mean (±standard deviation) scores of veterinary students’ responses to Likert‐type statements on artificial intelligence in veterinary public health education (*n* = 60).

	Strongly disagree *N* (%)	Disagree *N* (%)	Undecided *N* (%)	Agree *N* (%)	Strongly agree *N* (%)	Mean ± SD
The use of AI in veterinary medicine is increasingly causing legal and ethical conflicts.	3 (5.1)	11 (18.3)	18 (30.0)	23 (38.3)	5 (8.3)	2.73 ± 1.02
I think AI will reduce the need for veterinarians.	16 (26.7)	22 (36.7)	12 (20.0)	8 (13.3)	2 (3.3)	3.70 ± 1.10
I think artificial intelligence can be used as an educational tool in veterinary public health education.	3 (5.0)	6 (10.0)	13 (21.7)	27 (45.0)	11 (18.3)	2.38 ± 1.05
I plan my career according to developments in AI.	9 (15.0)	27 (45.0)	16 (26.7)	7 (11.6)	1 (1.7)	3.60 ± 0.94
I can apply artificial intelligence programmes about foodborne diseases.	2 (3.3)	2 (3.3)	11 (18.4)	39 (65.0)	6 (10.0)	2.25 ± 0.81

*Note*: Likert‐type scaling ranges from 1 = ‘strongly agree’ to 5 = ‘strongly disagree’. Therefore, a high average score indicates a low level of agreement (i.e., disagreement) with the relevant statement.

### 3.2 Qualitative Findings (Student Perspectives on AI in Education)

The open‐ended question asked students to evaluate AI in VPH education in terms of benefits, risks and metaphors. As a result of the analysis, codes were determined based on the participants’ evaluations of AI. The codes reflect both positive and negative perspectives of the students on the role of AI in education. These codes are categorized as follows:

*Positive codes*: Useful, quick access, practical, increasing use, alternative to lecture, time‐saving, general knowledge, tireless teacher, increased reliability in the food sector.

*Negative codes*: Lack of detail, human qualification, need for maximum knowledge, human exclusion, creates unemployment, feeling of inadequacy, intimidating, misinforming, misleading, dulling research/talent, providing false expertise, ethical violation, lack of experience.

**FIGURE 3:**
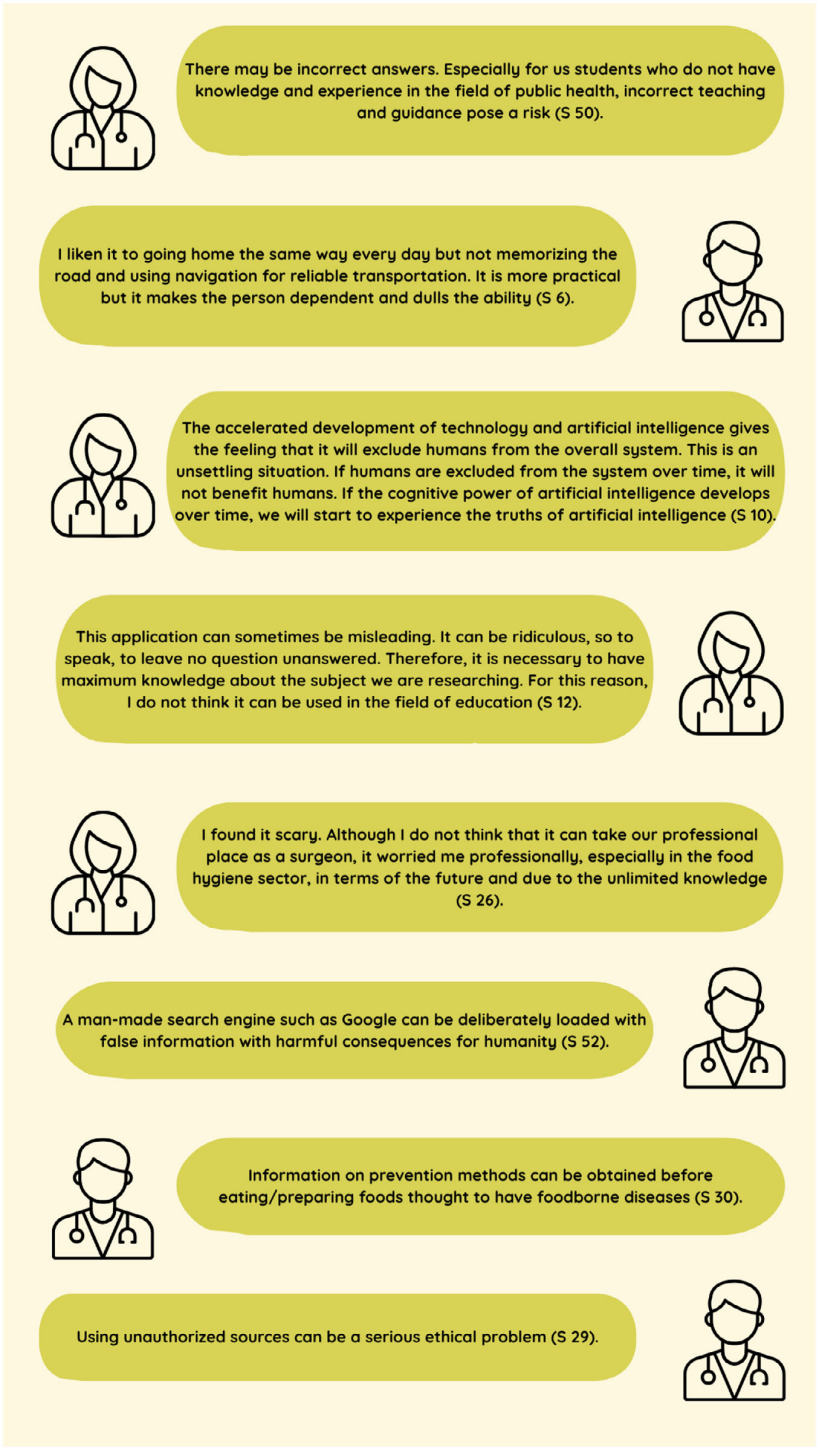
Veterinary students’ responses to open‐ended questions about artificial intelligence in veterinary public health education.

While some students stated that AI can be useful in acquiring technical information on public health, such as learning which foods contain pathogens and possible clinical findings (S50), and can be a consultable tool for preventive measures for foodborne diseases (S30), others pointed out that it can lead to misleading teaching (S12), especially for students with limited knowledge and experience in the field of public health, and emphasized the risk factors such as providing misinformation, making routing errors and causing weakening of knowledge‐based competence (S12, S50). It was also noted that over‐reliance on AI can dull individual research and thinking skills over time. One of the participants described this situation with the metaphor *‘I liken it to going home the same way every day but not memorizing the route and relying on navigation for reliable transportation. It is more practical but it makes one dependent and dulls the ability’ (S6)*. Some participants described AI with the metaphor of ‘a tireless teacher’ (S14), while others described it as ‘a frightening system’ (S26), ‘an incomplete advisor’ (S27) or ‘a mechanism that excludes humans’ (S10). Some participants expressed concerns that non‐veterinarians might use the information provided by AI to comment on the profession and that this would damage the image of the profession (S47). Some representative student views are illustrated in Figure 3.

## 4 Discussion

In this study, two main aspects were evaluated: anxiety levels regarding AI use and student perceptions of AI. First of all, factor analysis was performed. The Bartlett's test of sphericiy value was significant (*p* < 0.05) and the KMO value was greater than 0.06, indicating that the sample size was sufficient for EFA and the data set was suitable for modelling. As a result of the EFA, the rate of explaining the total variance of the AIAS scale was found to be 71.9%. This rate is a very strong indicator for the structural validity of the scale. The level of total variance explanation is a parameter that shows the degree of explanation of the scale and 50% and above is considered as an acceptable threshold in the literature (Yang et al. 2022; Fabrigar and Wegener 2012). Given the variance explanation rate of 71.9%, the AIAS appears to be a consistent and comprehensive instrument for measuring AI‐related anxiety among veterinary students. In addition, the fact that the Cronbach's *α* value of the scale was above 0.90 showed that its reliability was high (Özdamar 2002), that is, the AIAS was applied reliably. When the scores obtained from the scales were examined, it was seen that the general anxiety levels of veterinary students towards AI were ‘moderate’ in both cases (Akkaya et al. 2021) before (43.73) and after (42.27) AI‐supported VPH education. These results reveal that veterinary students feel a certain level of anxiety and uncertainty about the effects of AI technology on their professional future, but they do not have a high level of threat perception. Similarly, in studies conducted with veterinary students, it has been reported that students have a positive approach to AI (Busch et al. 2024; Yerlikaya and Küçükaslan 2024). Furthermore, the lack of significant differences in AIAS scores (*p* > 0.05) pre‐ and post‐training indicates that short‐term AI exposure is insufficient to alter general anxiety levels. Similarly, studies have reported that long‐term applications and experiences are more effective in reducing general anxiety towards AI than short‐term knowledge acquisition. In particular, students’ repetitive communication with AI allows them to get to know this technology more closely and thus contributes to an increase in confidence in technology and a decrease in anxiety levels (Wang and Wang 2019; Zawacki‐Richter et al. 2019; Sousa and Flay 2024). In line with these results, it can be said that in order to reduce AI anxiety in students, it is necessary to support AI education with long‐term and structured programmes, to enable students to interact more with AI, and to integrate AI literacy into the veterinary medicine curriculum.

In parallel with this, the fact that 72.9% of the participants in the study stated that the AI course should be added to the education curriculum indicates that veterinary students are open to technological developments and see this technology as a permanent and important tool in their professions. This view is also supported by the fact that 75% of the students stated that they already use AI tools ‘occasionally’ or ‘constantly’. This can be interpreted as an expectation that AI is already used by students and that it should be supported by formal education. These results also coincide with the literature (Yerlikaya and Küçükaslan 2024; Sousa and Flay 2024). In particular, the use of AI in veterinary medicine practice, such as the interpretation of radiographic images, clinical decision‐making and scientific research support, makes it necessary for students to have knowledge about AI and to be able to use AI effectively (Appleby and Basran 2022; Sobkowich 2025). At this point, as emphasized in the report published by the European Coordination Committee on Veterinary Training (ECCVT 2020) (European Coordination Committee on Veterinary Training (ECCVT) 2025), in order to ensure the safe, ethical and effective use of AI, both technological infrastructure should be developed and approaches that will enable students to approach these technologies critically and consciously should be integrated into the curriculum.

The fact that 63.3% of the participants responded positively to the question *‘I think AI can be used as an educational tool in VPH education’* and 75% of the participants responded positively to the question *‘I can apply AI programmes about foodborne diseases’* indicates that students see AI as a functional tool especially in areas such as public health, food safety and epidemiology. This shows that students are willing to use the power to access and analyse information with AI and are open to adopting AI in their education processes. These findings align with the objectives of the European Union‐funded SUSA project, which aims to enhance the digital competencies of healthcare professionals through AI‐based educational models (University of Oulu 2025). Aiming to improve the digital skills of health professionals, SUSA provides an infrastructure support for the effective use of AI‐based education models in health fields such as veterinary medicine. Therefore, the positive opinions of the students about the VPH course show the applicability of digital health projects created in this way for veterinary medicine education. In addition, foodborne diseases cause outbreaks of varying magnitudes around the world from time to time and significantly affect public health and social economy. Most foodborne diseases are zoonotic in nature and detection of pathogenic organisms and prevention methods are among the most important issues for food safety. AI has recently become an effective technique for predicting foodborne pathogens. One study aimed to create and use innovative molecular tools supported by AI to investigate foodborne diseases and predicted that it would help to better understand the aspects leading to the increase of these infections (Kumar et al. 2024; İncili et al. 2020). In another study (Wang et al. 2021), an AI‐assisted classification model was created for *Salmonella*, Norovirus, *Escherichia coli* and *Vibrio parahaemolyticus*, which are among the most important foodborne pathogens, and 69% accuracy was determined. In another study, an AI‐supported smart food science strategy was envisaged and it was thought that AI would make a promising contribution to models that could revolutionize food safety and foodborne disease research by providing diagnostic models that accelerate the analysis and screening of harmful components, toxins and pathogens in foods (Shangguan et al. 2025). In a study on the integration of AI technology into food safety management systems, it was concluded that AI is inspiring for food companies to bring the latest technologies to their operations and play a guiding role in food safety (Kudashkina et al. 2022). In a study investigating the simultaneous discrimination of five common foodborne pathogens (*Campylobacter jejuni, Escherichia coli, Listeria innocua, Salmonella typhimurium* and *Staphylococcus aureus)* by AI‐assisted hyperspectral microscopic imaging (HMI), the AI‐based system was found to be an effective tool for the identification of foodborne pathogens with an accuracy of 92.9% (Kang et al. 2021). In another study investigating AI‐assisted detection of foodborne pathogens, a detection method using AI transcoding (SMART) for rapid, sensitive and multiplex profiling of pathogens was developed, and it was observed that multiple bacteria were detected rapidly and simultaneously in < 10^2^ CFU/mL egg samples without DNA amplification and in agreement with standard microbiological and genotypic methods (Feng et al. 2023).

The results of the study show that most of the prospective veterinarians (63.4%) disagree with the statement *‘I think that AI will reduce the need for veterinarians’*. This suggests that students perceive AI as a tool that supports and complements their professions rather than seeing it as a direct threat. In the literature, it has been emphasized that professionals working in the field of health have a similar approach and that AI plays an auxiliary role by alleviating the burden of the human factor in clinical decision processes, but that humans will remain an indispensable element in dimensions such as human–animal interaction, privacy and ethical responsibilities (Yerlikaya and Küçükaslan 2024; Davenport and Kalakota 2019; Esteva et al. 2019). In addition, the majority of the participants in the study (60%) stated that they did not direct their careers in the direction of AI. This may be due to several reasons. The first reason may be that students see AI as a supportive tool for their professions rather than a threat to their professions due to the reasons mentioned above. The second reason may be that they are not aware of the impact of AI on the veterinary medicine profession and therefore do not internalize AI sufficiently. Further studies are needed to clarify this issue.

The increasing use of AI in veterinary medicine raises important legal and ethical issues. Studies have reported that the inclusion of AI applications in diagnosis and decision‐making processes may lead to legal uncertainties in terms of responsibility, accountability and data privacy. In addition, it is argued that by reducing direct human interaction with sick animals and their owners, it may weaken factors such as empathy and personal responsibility and may contradict principles such as justice and equality (Yerlikaya and Küçükaslan 2024; Esteva et al. 2019; Cohen and Gordon 2022). In the current study, similarly, the fact that a significant portion of the participants (46.6%) thought that the use of AI in veterinary medicine would cause more and more legal and ethical conflicts shows that there are serious concerns about the ethical and legal dimensions of AI among prospective veterinarians. This can be interpreted as the fact that the legal basis and ethical guidance on AI have not developed in parallel with the developing technology, which increases professional concerns. Therefore, it should be emphasized that supporting the safe use of AI with professional guidance and legal frameworks is critical for the integration of AI into veterinary medicine.

On the other hand, qualitative findings revealed a dual perception structure among veterinary students regarding AI: While students viewed AI as a practical and time‐saving learning tool, they also expressed concerns about misinformation, dependency and potential threats to their professional identity. This dichotomy suggests that perceptions and concerns may develop differently, and structured, long‐term educational interventions may be necessary to both reduce anxiety and ensure the effective integration of AI into veterinary education. Similar ambivalent attitudes have also been reported in medical and health education studies, where students recognized AI as a valuable support for clinical decision‐making yet remained cautious about its reliability and ethical implications (Chan and Zary 2019). In line with our findings, recent research in dental and nursing education has shown that although students appreciate the efficiency and accessibility of AI tools, they also worry about professional devaluation and loss of critical thinking skills (Sharab et al. 2024; Aydın et al. 2025). Overall, these findings indicate that veterinary students, similar to their peers in other health professions, approach AI with both optimism and caution.

## 5 Conclusion

The study revealed that students’ anxiety regarding AI remained at a moderate level, and short‐term AI‐supported training did not induce a significant change in these levels. Students’ perceptions of AI in VPH education exhibited a multidimensional structure, balancing positive attributes—such as rapid information access and practicality—against potential risks like ethical concerns and the erosion of expertise. It was also found that students were open to adopting AI as a supportive tool in education. In order to effectively integrate AI‐supported education methods into veterinary education, it is very important to make a pedagogically careful planning by taking into account students' attitudes, anxiety levels and technological competencies. The first step to be taken for this is to provide students with AI literacy. In addition, it can be said that balancing the opportunities offered by AI with the risks it carries is of great importance in terms of integrating this technology into education in a healthy way.

## 6 Limitations

This study has several limitations that should be acknowledged. First, the study was conducted at a single veterinary faculty, which may limit the generalizability of the findings. Second, the wide variation in participants’ prior AI experience may have influenced their perceptions and anxiety scores. Third, the short‐term nature of the training limited the ability to capture long‐term changes in anxiety or perceptions. Finally, the data were based on self‐reported measures, which may be subject to bias. Future research should therefore include larger, multi‐institutional samples, longitudinal designs and objective performance outcomes.

## Author Contributions

*Conception, design, data collection, writing, reviewing and editing*: Selçuk Alan, Gönül D. Büyük and Seda Ç. Alan. *Analysis and interpretation*: Selçuk Alan and Seda Ç. Alan. All authors are responsible for the accuracy and integrity of all aspects of the work.

## Funding

The authors received no specific funding for this work. The Open Access publication fee (Article Processing Charge, APC) was covered through the national Open Access agreement between the Scientific and Technological Research Council of Türkiye (TÜBİTAK) and Wiley.

## Ethics Statement

This study was approved by the Non‐Interventional Research Ethics Committee of the Faculty of Health Sciences, Kafkas University (Approval Date: 31 December 2024; Approval No: 2024/10). In addition, since the study was conducted with veterinary students, the necessary institutional permission was also obtained from the Dean's Office of the Faculty of Veterinary Medicine, Kafkas University.

## Conflicts of Interest

The authors declare no conflicts of interest.

## Data Availability

The data supporting this study's findings are available from the corresponding author upon reasonable request.
